# Effects of Lubiprostone, an Intestinal Secretagogue, on Electrolyte Homeostasis in Chronic Idiopathic and Opioid-induced Constipation

**DOI:** 10.1097/MCG.0000000000001385

**Published:** 2020-06-19

**Authors:** Satish S.C. Rao, Peter Lichtlen, Sepideh Habibi

**Affiliations:** * Augusta University Medical Center, Augusta, GA; † Mallinckrodt Pharmaceuticals, Zug, Switzerland; ‡ Mallinckrodt Pharmaceuticals, Rockville, MD

**Keywords:** lubiprostone, electrolyte homeostasis, irritable bowel syndrome with constipation, opioid-induced constipation, chronic idiopathic constipation

## Abstract

To assess short-term and long-term effects of lubiprostone, a type-2 chloride channel activator, on electrolyte homeostasis.

**Background:**

Conventional laxatives are associated with electrolyte imbalances. Lubiprostone is a type-2 chloride channel activator approved for treating chronic idiopathic constipation (CIC), opioid-induced constipation (OIC), and constipation-predominant irritable bowel syndrome in women. It induces intestinal fluid secretion, possibly affecting water and electrolyte homeostasis. We investigated short-term and long-term effects of lubiprostone on electrolyte, blood urea nitrogen (BUN), and creatinine levels using pooled data from CIC and OIC patients.

**Study:**

Data were pooled from 10 CIC and OIC studies—6 double-blind, randomized, placebo-controlled studies and 4 open-label, long-term studies. Total duration of lubiprostone exposure was from 3 weeks (short-term: CIC, 3 to 4 wk; OIC, placebo-controlled, 12 wk) to 48 weeks (long-term: CIC, 24 to 48 wk; OIC, 48 wk). Sodium, chloride, potassium, magnesium, BUN, and creatinine levels were examined at baseline and final assessment.

**Results:**

Overall, 3209 patients were assessed. In the double-blind, placebo-controlled studies, there were no clinically meaningful differences in levels of electrolytes, BUN, and creatinine between lubiprostone and placebo groups, and in changes from baseline levels with long-term use of lubiprostone. Analyses of shifts in laboratory values (low/normal/high) at baseline and final assessment showed minimal effects on electrolytes, BUN, and creatinine.

**Conclusions:**

Lubiprostone did not cause clinically meaningful electrolyte imbalances or affect markers of renal function in either the short-term or long-term treatment of CIC or OIC.

Chronic constipation has a worldwide prevalence of ~14% and is the most common gastrointestinal adverse event (AE) reported by opioid users.^1^,^2^ Common treatments include dietary changes, fiber, and conventional laxatives.^3^ Laxatives improve gastrointestinal transit by inducing an influx of water to the intestinal lumen. However, the promotion of intestinal fluid secretion by osmotic saline laxatives such as magnesium hydroxide, sodium phosphates, and magnesium citrate may alter electrolyte homeostasis and induce serum electrolyte imbalance in patients with renal insufficiency or cardiac dysfunction.^4^,^5^

Management of chronic constipation has advanced in recent years because of the emergence of multiple novel agents, such as guanylate cyclase C receptor activators, 5-hydroxytryptamine receptor 4 (5-HT4) receptor agonists, and chloride channel activators.^6^ Lubiprostone is a type-2 chloride channel (ClC-2) activator approved in the United States for the treatment of chronic idiopathic constipation (CIC) in adults, opioid-induced constipation (OIC) in adults with chronic noncancer pain, and constipation-predominant irritable bowel syndrome (IBS-C) in women aged 18 years old and above. In contrast to osmotic laxatives, lubiprostone increases intestinal fluid in a physiological manner by first inducing chloride secretion from intestinal epithelial cells, followed by passive paracellular sodium secretion leading ultimately to water influx to the intestinal lumen.^7^ Although individual clinical trials of lubiprostone showed no effects on serum electrolytes, it is possible that important but subtle shifts in electrolytes may not have been identified in these studies. Therefore, we designed this large post hoc, pooled analysis to evaluate the potential effect of this intestinal secretagogue on electrolyte homeostasis.

The objective of this pooled analysis was to assess the short-term and long-term effects, including any possible safety concerns, of lubiprostone 24 mcg twice daily (BID) on electrolyte homeostasis by examining the serum electrolyte levels, blood urea nitrogen (BUN), and creatinine in patients participating in multiple dose-ranging controlled phase 3 and open-label trials in patients with CIC and OIC.^6^

## METHODS

### Study Selection for Pooled Analysis

We identified all studies conducted by Sucampo up until the time of manuscript preparation and included those that used the highest currently approved lubiprostone dose (24 mcg BID). These studies were selected for this analysis under the premise that any potential issues with electrolyte imbalance would be most apparent with this regimen. IBS-C studies were excluded from this analysis because the patients were exposed to a lower (8 mcg BID) dose. Short-term studies (up to 12 wk) were included because they had placebo-controlled arms, while the long-term trials allowed the analysis of lubiprostone for exposure of up to 1 year. A total of 10 lubiprostone trials with 24 mcg BID dosing, 6 in CIC and 4 in OIC, were identified. Data were derived from a pooled analysis of these trials and provided to all of the authors for presentation and discussion here.

### Original Study Design and Patients

**FIGURE 1:**
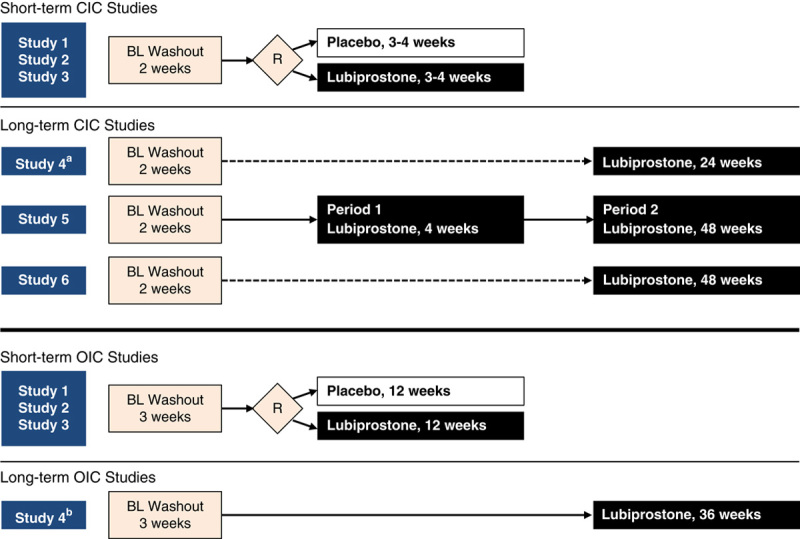
Design of the studies. ^a^Extension study for CIC study 1 and newly enrolled patients (following a 2-week BL washout period). A total of 308 patients were assessed in CIC study 4 (newly assessed, n=117; previously received placebo, n=107; previously received lubiprostone, n=84). ^b^Extension study for OIC study 1 and study 2. BL indicates baseline; CIC, chronic idiopathic constipation; OIC, opioid-induced constipation; R, randomization.

Of the 6 trials in CIC, 3 were double-blind, randomized, placebo-controlled short-term (3 to 4 wk) studies, and 3 were open-label long-term (24 or 48 wk) studies (Fig. 1).^8^–^13^ Of the 4 OIC trials, 3 were double-blind, randomized, placebo-controlled 12-week studies, and 1 was a 36-week open-label extension study.^14^–^17^ The pooled study population across the 10 trials consisted of 3209 patients with OIC or CIC who were randomized for assessment (Table 1). Among these, a total of 3176 patients were evaluated and included in this analysis. CIC short-term and long-term studies included 544 and 878 patients, respectively, whereas OIC placebo-controlled and open-label long-term studies included 1315 and 439 patients, respectively. The methods described below reflect those utilized in the original studies that were selected for this analysis.

**TABLE 1:** Study Details and Summary of Patient Disposition

	CIC Studies (N=1429), n (%)						OIC Studies (N=1780), n (%)			
Variables	Short-term			Long-term			Placebo-Controlled			Long-term
Publication information	Study 1: Johanson et al^8^	Study 2: Johanson et al^9^	Study 3: Barish et al^10^	Study 4: Johanson et al^11^	Study 5: Fukudo et al^13^	Study 6: Lembo et al^12^	Study 1: Jamal et al^15^	Study 2: Cryer et al^14^	Study 3: Jamal et al^16^	Study 4: Spierings et al^17^
Design	Double-blind, randomized, placebo-controlled	Double-blind, randomized, placebo-controlled	Double-blind, randomized, placebo-controlled	Open-label safety	Randomized withdrawal and open-label safety	Open-label safety	Double-blind, randomized, placebo-controlled	Double-blind, randomized, placebo-controlled	Double-blind, randomized, placebo-controlled	Open-label extension
Primary endpoint(s)	Daily average no. of SBMs/week	SBM frequency at week 1	SBM frequency at week 1	AEs, lab values, vital signs, PE	AEs, lab values, vital signs, PE	AEs, lab values, vital signs, PE	Change in SBM frequency at week 8	Change in SBM frequency at week 8	Overall SBM response	AEs, lab values, vital signs, PE, change in SBM frequency/month
No. of patients assessed	65 (100)	244 (100)	237 (100)	308 (100)	325 (100)	250 (100)	443 (100)	451 (100)	447 (100)	439 (100)
No. of treated patients	65 (100)	242 (99.2)	237 (100)	306 (99.4)	324 (99.7)	248 (99.2)	439 (99.1)	437 (96.9)	439 (98.2)	439 (100)
No. of patients who completed the study	55 (84.6)	224 (91.8)	206 (86.9)	165 (53.6)	153 (47.1)	127 (50.8)	306 (69.1)	305 (67.6)	353 (79)	286 (65.1)
Reasons for discontinuation										
AE	7 (10.7)	10 (4.1)	16 (6.8)	60 (19.5)	68 (20.9)	33 (13.2)	23 (5.2)	14 (3.1)	15 (3.4)	23 (5.2)
Protocol violation	0 (0)	0 (0)	0 (0)	0 (0)	4 (1.2)	1 (0.4)	—	—	—	—
Subject voluntary withdrawal	0 (0)	4 (1.6)	1 (0.4)	14 (4.5)	22 (6.8)	23 (9.2)	27 (6.1)	39 (8.6)	27 (6.0)	47 (10.7)
Lack of efficacy	3 (4.6)	3 (1.2)	7 (3.0)	49 (15.9)	58 (17.8)	44 (17.6)	12 (2.7)	7 (1.6)	11 (2.5)	15 (3.4)
Lost to follow-up	0 (0)	3 (1.2)	5 (2.1)	16 (5.2)	15 (4.6)	17 (6.8)	19 (4.3)	24 (5.3)	8 (1.8)	31 (7.1)
DC of opioids	—	—	—	—	—	—	6 (1.4)	3 (0.7)	3 (0.7)	7 (1.6)
Noncompliance	—	—	—	—	—	—	7 (1.6)	6 (1.3)	7 (1.6)	9 (2.1)
Investigator decision	—	—	—	—	—	—	5 (1.1)	4 (0.9)	4 (0.9)	5 (1.1)
Did not meet entry criteria	0 (0)	0 (0)	0 (0)	1 (0.3)	0 (0)	0 (0)	—	—	—	—
Sponsor request	—	—	—	—	—	—	4 (0.9)	5 (1.1)	5 (1.1)	—
Other	0 (0)	0 (0)	2 (0.8)	3 (1.0)	5 (1.5)	5 (2.0)	34 (7.7)	44 (9.8)	14 (3.1)	13 (3.0)

AE indicates adverse event; CIC, chronic idiopathic constipation; DC, discontinuation; lab, laboratory; OIC, opioid-induced constipation; PE, physical examination; SBM, spontaneous bowel movement.

#### Patient Inclusion/Exclusion Criteria for Original Studies

Eligible patients were aged 18 years old and above who had constipation with <3 spontaneous bowel movements per week and at least 1 of 3 associated symptoms: hard or very hard stools, sensation of incomplete evacuation, or moderate to very severe straining at defecation. All patients were required to have discontinued use of laxative and stool softeners throughout the study period (except in cases of rescue use). Patients were excluded from the studies if they had impaired renal function or renal abnormalities. Eating disorders, including bulimia, were not criteria for excluding patients from any of the trials. OIC patients were required to be treated with an opioid for ≥30 days for chronic noncancer pain, and for patients with a history of chronic constipation (≥90 d), constipation symptoms had to be exacerbated by the initiation of opioid treatment.

Exclusion criteria for the CIC trials included mechanical obstruction or pseudo-obstruction, known or suspected organic disorders of the large or small intestine, prior bowel resection or abdominal surgical procedure during the 3 months before the start of the study, cancer within the last 5 years, or the presence of systemic disease.^9^ Key exclusion criteria for the OIC trials included the use of opioids for pain due to cancer, unwillingness to refrain from use of laxatives during study participation, abdominal pain, scleroderma, unexplained weight loss within 90 days before screening, drug addiction, and the presence of mechanical bowel obstruction, organic intestinal disorders, constipation unrelated to opioid use, endocrine disorders, or any disease or disorder that could interfere with conduct of the studies.^14^,^18^

### Outcomes and Assessment

Serum sodium, chloride, potassium, and magnesium, BUN, and creatinine levels were determined at baseline and final assessment at the end of study period or early discontinuation. In the individual studies, biochemical assays were performed by an accredited laboratory facility, and the results were reviewed by the trial investigators. Electrolyte levels were categorized by laboratory-defined lower and upper limits of normal as “low,” “normal,” or “high.”

### Compliance With Ethical Standards

The 10 lubiprostone studies involved human participants and were conducted according to the principles described in the Declaration of Helsinki, International Conference on Harmonization Harmonized Tripartite Guideline for Good Clinical Practice. Patient consent was obtained before commencement of any study procedures in each study.

### Statistical Analyses

The baseline period for each selected study was defined as 14 days before randomization or intake of first study medication, and baseline values for all studies were calculated as mean values from that period. Independent determinations of serum electrolytes were made before intake of the first study dose, at screening, and at baseline.

The safety evaluable population for all studies was made up of all study participants who took at least 1 dose of study medication; safety data from the date of first dose of study medication through 7 days past treatment termination were included in the safety analyses. AEs emerging during this safety window were considered as treatment-emergent. In OIC placebo-controlled studies (study 2 and study 3), patients who received placebo in the randomized phase and participated in the long-term study (study 4) were included in both the placebo and lubiprostone groups and were counted twice in the total number of patients. Patients who received lubiprostone in OIC placebo-controlled studies (study 2 and study 3) and then participated in the long-term study (study 4) factored only once in the lubiprostone group and thus were counted once in the total number of patients.

Where appropriate, short-term and long-term results were combined from respective studies using descriptive statistics (n, mean, median, SD, minimum and maximum values). Mean difference was used to pool results for serum electrolytes and renal function markers. For short-term studies, comparisons in mean change from baseline were made between the placebo and lubiprostone groups. For long-term studies, only within-group mean change from baseline was calculated. AEs were summarized as the number and percentage of patients. One-way analysis of variance was used to calculate the 95% confidence intervals and *P*-values. Statistical analyses, data summaries, and listings were provided using version 8.2 or later of SAS software (Cary, NC).

The handling of missing information varied by data type. Missing days or months for historical data, such as medical history, were generally not imputed since this would have had little effect on the analysis. However, data related to AEs were imputed so that conservative (worst-case) estimates could be made based on the available information.

## RESULTS

### Patients

The pooled study population across the 10 trials consisted of 3209 patients with OIC or CIC who were randomized for assessment (Table 1). Among these, 3176 patients were evaluated and 2180 completed the trials. Age and race distributions were similar across all groups at baseline (Table 2). A greater percentage of women than men participated in each of the studies, especially in the CIC studies. Females represented 86% to 90% and 60% to 63% of CIC and OIC patients, respectively (Table 2).

**TABLE 2:** Baseline Patient Characteristics

	CIC			OIC		
	Short-term		Long-term	Placebo-controlled		Long-term
Characteristics	Placebo (n=273)	Lubiprostone (48 mcg) (n=271)	Lubiprostone (48 mcg) (n=878)	Placebo (n=652)	Lubiprostone (48 mcg) (n=663)	Lubiprostone (48 mcg) (n=439)
Sex n (%)						
Male	27 (9.9)	32 (11.8)	121 (13.8)	244 (37.4)	247 (37.3)	176 (40.1)
Female	246 (90.1)	239 (88.2)	757 (86.2)	408 (62.6)	416 (62.7)	263 (59.9)
Mean age (SD) (y)	47.2 (13.1)	47.4 (12.2)	51.1 (13.64)	50.5 (11.5)	50.3 (9.7)	49.8 (9.9)
Race n (%)						
White	220 (80.6)	224 (82.7)	764 (87.0)	539 (82.9)	545 (82.2)	367 (83.8)
Black	27 (9.9)	24 (8.9)	64 (7.3)	90 (13.8)	98 (14.8)	55 (12.6)
Asian	3 (1.1)	4 (1.5)	6 (0.7)	5 (0.8)	5 (0.8)	3 (0.7)
Other	23 (8.4)	19 (7.0)	44 (5.0)	16 (2.5)	12 (1.8)	13 (3.0)

Values are represented as n (%).

CIC indicates chronic idiopathic constipation; OIC, opioid-induced constipation.

### Electrolyte, BUN, and Creatinine Concentrations

Pooled analyses of electrolyte levels or markers of renal function were performed separately for each of the 4 study designs (short-term CIC studies, long-term CIC studies, short-term OIC studies, and the long-term OIC study). Mean and individual values at baseline and final assessment for serum electrolytes, BUN, and creatinine were within normal ranges in patients with CIC or OIC (Table 3).

**TABLE 3:** Shift of Baseline Serum Electrolytes and Renal Marker Category During Treatment Period*

		CIC			OIC		
		Short-term		Long-term	Placebo-controlled		Long-term
Parameters	Final Assessment	Placebo (n=273)	Lubiprostone (48 mcg) (n=271)	Lubiprostone (48 mcg) (n=878)	Placebo (n=652)	Lubiprostone (48 mcg) (n=663)	Lubiprostone (48 mcg) (n=439)
Sodium	Low	4/261	2/255	10/793	12/575	20/583	11/410
	Normal	256/261	251/255	783/793	563/575	563/583	398/410
	High	1/261	2/255	0/793	0/575	0/583	1/410
Potassium	Low	9/257	4/249	17/780	18/579	20/588	13/413
	Normal	246/257	241/249	756/780	555/579	566/588	396/413
	High	2/257	4/249	7/780	6/579	2/588	4/413
Chloride	Low	2/263	0/254	7/796	15/574	10/585	8/403
	Normal	261/263	252/254	785/796	556/574	572/585	393/403
	High	0/263	2/254	4/796	3/574	3/585	2/403
Magnesium	Low	0/254	0/245	2/789	1/587	1/601	1/416
	Normal	254/254	241/245	779/789	582/587	596/601	415/416
	High	0/254	4/245	8/789	4/587	4/601	0/416
BUN	Low	0/230	0/225	0/798	3/578	3/591	3/416
	Normal	228/230	224/225	783/798	565/578	585/591	406/416
	High	2/230	1/225	15/798	10/578	3/591	7/416
Creatinine	Low	0/266	0/258	1/807	13/546	14/552	0/418
	Normal	265/266	257/258	800/807	524/546	532/552	415/418
	High	1/266	1/258	6/807	9/546	6/552	3/418

*Table entries show the number of patients moving from “normal” baseline value for the indicated parameter to the indicated value at the final assessment.

BUN indicates blood urea nitrogen; CIC, chronic idiopathic constipation; OIC, opioid-induced constipation.

**FIGURE 2:**
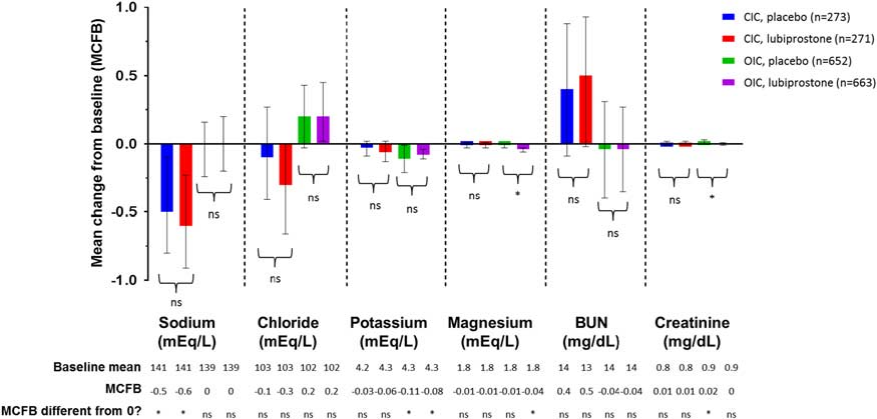
Serum electrolytes and markers of renal function in short-term CIC and placebo-controlled OIC studies. *P<0.05. Parentheses denote comparison between placebo and lubiprostone groups. The histogram represents the mean change from baseline at final assessment; error bars represent 95% confidence intervals. BUN indicates blood urea nitrogen; CIC, chronic idiopathic constipation; ns, not statistically significant; OIC, opioid-induced constipation.

**FIGURE 3:**
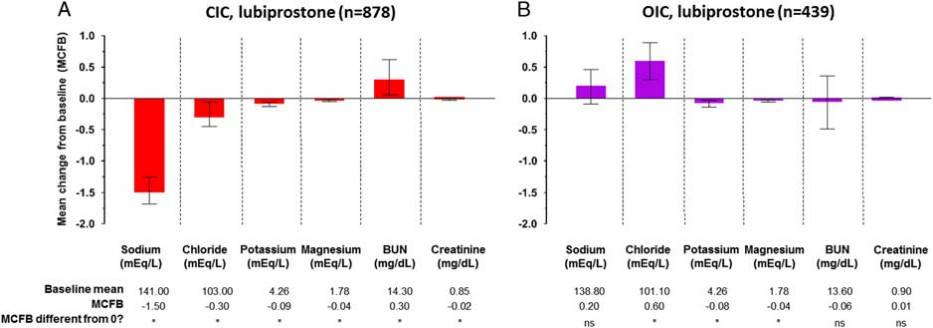
Serum electrolytes and markers of renal function in long-term CIC studies (A) and long-term OIC studies (B). *P<0.05. The histogram represents the mean change from baseline at final assessment; error bars represent 95% confidence intervals. BUN indicates blood urea nitrogen; CIC, chronic idiopathic constipation; ns, not statistically significant; OIC, opioid-induced constipation.

There were no apparent clinically meaningful effects of lubiprostone on average serum sodium, chloride, potassium, and magnesium, BUN, and creatinine levels in patients with CIC or OIC after short-term or long-term treatment (Figs. 2, 3). There were statistically significant (*P*<0.05) differences found in comparisons between OIC placebo versus OIC lubiprostone groups in mean change from baseline levels for magnesium (−0.01 vs. −0.04 mEq/L) and creatinine (0.02 vs. 0 mg/dL) but those were within normal clinically acceptable ranges (Fig. 2). The statistically significant changes from baseline in long-term treatment with lubiprostone were also within normal clinically acceptable ranges: −1.50 mEq/L for sodium, 0.3 mg/dL for BUN and ranging from −0.30 to 0.6 mEq/L for chloride, potassium, magnesium, and creatinine (Fig. 3).

No important clinical trends were observed for shifts in electrolytes, BUN, or creatinine from one category (low, normal, and high) to another category after lubiprostone treatment relative to placebo in the pooled population. Among the patients who were in the “normal” range for serum electrolytes, BUN, or creatinine at baseline, very few (<4.3%) shifted outside of the “normal” range at the final assessment in either the short-term or long-term studies (Table 3). In the short-term studies, the numbers of patients who shifted from “normal” at baseline to “low” or “high” at the final assessment for these parameters were comparable between the placebo and lubiprostone groups.

### Safety

Treatment-related AEs reported were numerically greater among patients in the lubiprostone group than in the placebo group [129 (47.6%) vs. 52 (19.0%)] in the short-term CIC studies, while 481 (54.8%) patients reported treatment-related AEs in the long-term CIC studies. The same pattern was observed in the placebo-controlled OIC studies with 204 (30.8%) patients in the lubiprostone group and 132 (20.2%) patients in the placebo group reporting treatment-related AEs. A numerically lower number [108 (24.6%)] reported treatment-related AEs in the long-term OIC study. No treatment-related serious adverse events (SAEs) were reported in the short-term CIC or long-term OIC studies; however, 2 patients (0.2%) in the long-term CIC studies and 3 patients (0.5%) in the placebo group of the placebo-controlled OIC studies reported treatment-related SAEs. The treatment-related SAEs in the long-term CIC studies were diarrhea and congenital clubfoot in the offspring of a study participant, both rated as possibly related to study drug by the investigator.

The proportion of patients reporting at least 1 severe AE was comparable between the placebo and lubiprostone groups in OIC placebo-controlled studies (8.0% vs. 8.9%) and CIC short-term studies (5.1% vs. 7.7%). In long-term studies, 17.9% of CIC and 16.4% of OIC patients reported at least 1 severe AE.

In the short-term CIC studies, approximately 40% of patients younger than 65 years in the placebo group (39.8% in patients below 50 y and 40.7% in patients between 50 and 65 y) and 61.3% of patients 65 or older reported at least 1 AE. Across all active doses, 69.5% of patients younger than 50, 60.6% of patients between 50 and 65, and 47.1% of patients 65 or older reported at least 1 AE. In the long-term CIC cohort, 78.4% of patients younger than 50, 82.6% of patients between 50 and 65, and 74.2% of patients 65 or older reported at least 1 AE. In the OIC studies, patients were stratified into 2 groups by age: younger than 65 or 65 and older. The number of patients reporting at least 1 AE was comparable in the placebo and lubiprostone groups (52.8% vs. 57.8%, respectively) in patients younger than 65, and in patients 65 or older (49.3% and 53.3%, respectively) in the short-term studies. In the long-term OIC studies, the number of patients reporting at least 1 AE was higher but comparable across age strata in patients younger than 65 (71.3%) and 65 or older (74.2%). AEs indicating clinically significant changes in serum electrolytes, BUN, and creatinine were infrequent (≤0.9%) in patients treated with placebo or lubiprostone (Table 4). The only AEs relating to serum electrolyte changes that occurred in ≥3 patients were hypokalemia and increased serum urea concentration (Table 4).

**TABLE 4:** Adverse Events Potentially Indicative of Changes in Serum Electrolytes and Markers of Renal Function

		Incidence, n (%)	
Indication and Study Duration	Adverse Event	Placebo	Lubiprostone
CIC			
Short-term studies		(n=273)	(n=271)
	Hypokalemia	0 (0.0)	1 (0.4)
	Increased urinary nitrogen	0 (0.0)	1 (0.4)
Long-term studies		—	(n=878)
	Hypokalemia	—	1 (0.1)
	Hypophosphatemia	—	1 (0.1)
OIC			
Placebo-controlled studies		(n=652)	(n=663)
	Hyperkalemia	1 (0.2)	0 (0.0)
	Hypokalemia	1 (0.2)	2 (0.3)
	Hypomagnesemia	1 (0.2)	0 (0.0)
	Hyponatremia	0 (0.0)	1 (0.2)
	Blood calcium decreased	0 (0.0)	1 (0.2)
	Blood calcium increased	0 (0.0)	1 (0.2)
	Blood chloride decreased	1 (0.2)	1 (0.2)
	Blood creatinine increased	1 (0.2)	2 (0.3)
	Blood magnesium decreased	0 (0.0)	1 (0.2)
	Blood phosphorus decreased	1 (0.2)	0 (0.0)
	Blood phosphorus increased	1 (0.2)	0 (0.0)
	Blood potassium decreased	4 (0.6)	3 (0.5)
	Blood potassium increased	2 (0.3)	1 (0.2)
	Blood sodium decreased	1 (0.2)	2 (0.3)
	Blood urea increased	1 (0.2)	2 (0.3)
Long-term studies		—	(n=439)
	Hypokalemia	—	1 (0.2)
	Hyponatremia	—	2 (0.5)
	Blood calcium decreased	—	1 (0.2)
	Blood calcium increased	—	2 (0.5)
	Blood magnesium decreased	—	2 (0.5)
	Blood magnesium increased	—	1 (0.2)
	Blood phosphorus increased	—	2 (0.5)
	Blood potassium decreased	—	4 (0.9)
	Blood potassium increased	—	2 (0.5)
	Blood sodium decreased	—	1 (0.2)
	Blood urea increased	—	3 (0.7)
	Blood creatinine increased	—	2 (0.5)

CIC indicates chronic idiopathic constipation; OIC, opioid-induced constipation.

## DISCUSSION

Chronic constipation is a life-long problem, and for many patients it requires long-term use of medication. Consequently, medications that are used to treat this disorder should demonstrate not only efficacy but also long-term safety. Efficacy and safety are particularly important for drugs such as osmotic laxatives or intestinal secretagogues that may affect or alter electrolyte homeostasis. Several studies have shown that dehydration and disturbances in salt and electrolytes are a concern when using osmotic saline laxatives such as magnesium hydroxide, sodium phosphates, and magnesium citrate in patients with CIC or OIC, especially those with cardiac dysfunction or renal insufficiency.^4^,^19^ In contrast to saline laxatives, lubiprostone specifically targets the intestinal chloride channel ClC-2 to induce ion flux across intestinal epithelium and subsequent water movement into the intestinal lumen.^7^,^20^ Electrolyte imbalance may also affect smooth muscle and nerve function, compromise bowel function and cause constipation.

Our pooled analysis of a large cohort of patients with CIC or OIC across 10 trials showed no evidence that administration of lubiprostone 24 mcg BID for up to 48 weeks caused electrolyte imbalance or altered markers of renal function compared with baseline or placebo. Also, to our knowledge, this is one of the largest pooled analyses of safety data for a drug that could potentially affect intestinal secretion of fluids and thereby alter electrolyte homeostasis.

The serum concentrations of sodium, chloride, and potassium did not change outside of normal laboratory values in both the short-term and long-term studies and in CIC and OIC patients.^21^ Mean changes from baseline in the levels of sodium, chloride, and potassium were no >1.5, 0.6, and 0.11 mEq/L, respectively, in any of the studies. Similarly, changes in the markers of renal function were within the normal laboratory values. BUN and creatinine levels did not change from baseline by >0.5 and 0.02 mg/dL, respectively, in any of the studies. These small shifts in electrolyte concentrations are well within the normal range and unlikely to have any meaningful clinical effects. Also, the data from long-term monitoring provide confidence that lubiprostone is safe and unlikely to cause clinically relevant electrolyte imbalances in patients.

There were some limitations to this pooled analysis. The results shown in this analysis were derived from a predominantly white patient population, with a mean age of approximately 50 years, and a majority of whom were female patients. Thus, based on these data, lubiprostone’s effect on electrolyte imbalances or affected markers of renal function can only be concluded in the population involved in these studies. However, in other reports, age, sex, and race have not been shown to affect lubiprostone efficacy.^22^ All of the study patients were ambulatory and free of significant comorbid illnesses, such as chronic renal failure or liver or heart failure. Thus, we cannot comment on the effects of lubiprostone in potential patients with these illnesses. In addition, this analysis may not be optimal to provide insight into the issue of risk of electrolyte imbalance associated with laxative abuse, since patients in clinical trials tend to comply with medication as dosed (given pill counts).^23^,^24^ These and other users require further long-term safety monitoring.

In conclusion, our large pooled analysis of over 3000 patients shows that the chloride channel activator lubiprostone did not change serum electrolyte levels in patients with OIC or CIC. The approved dose of lubiprostone 24 mcg BID did not alter serum sodium and potassium concentrations or affect markers of renal function, and our data suggest that electrolyte imbalance is unlikely with routine use of lubiprostone for up to 48 weeks in the management of patients with OIC or CIC
